# The Influence of Mineral Fertilizer Combined With a Nitrification Inhibitor on Microbial Populations and Activities in Calcareous Uzbekistanian Soil Under Cotton Cultivation

**DOI:** 10.1100/tsw.2001.301

**Published:** 2001-10-30

**Authors:** Dilfuza Egamberdiyeva, Muhiddin Mamiev, Svetlana K. Poberejskaya

**Affiliations:** Institute of Microbiology, Uzbek Academy of Sciences, Tashkent, Uzbekistan

**Keywords:** ammophos, cellulose decomposition, microorganisms, nitrification inhibitor, urea

## Abstract

Application of fertilizers combined with nitrification inhibitors affects soil microbial biomass and activity. The objective of this research was to determine the effects of fertilizer application combined with the nitrification inhibitor potassium oxalate (PO) on soil microbial population and activities in nitrogen-poor soil under cotton cultivation in Uzbekistan. Fertilizer treatments were N as urea, P as ammophos, and K as potassium chloride. The nitrification inhibitor PO was added to urea and ammophos at the rate of 2%. Three treatments—N_200_P_140_K_60_ (T1), N_200_ P_140 PO_K_60_ (T2), and N_200_ P_140 PO_K_60_ (T3) mg kg soil—were applied for this study. The control (C) was without fertilizer and PO. The populations of oligotrophic bacteria, ammonifying bacteria, nitrifying bacteria, denitrifying bacteria, mineral assimilating bacteria, oligonitrophilic bacteria, and bacteria group Azotobacter were determined by the most probable number method. The treatments T2 and T3 increased the number of oligonitrophilic bacteria and utilization mineral forms of nitrogen on the background of reducing number of ammonifying bacteria. T2 and T3 also decreased the number of nitrifying bacteria, denitrifying bacteria, and net nitrification. In conclusion, our experiments showed that PO combined with mineral fertilizer is one of the most promising compounds for inhibiting nitrification rate, which was reflected in the increased availability and efficiency of fertilizer nitrogen to the cotton plants. PO combined with mineral fertilizer has no negative effects on nitrogen-fixing bacteria Azotobacter and oligo-nitrophilic bacteria.

## INTRODUCTION

Nitrogen fertilizers in the form of urea are commonly applied in Uzbekistan in order to increase cotton yield in less fertile soils. The ammonia ($NO_3$) formed through nitrification of urea is susceptible to loss through leaching and may contribute to $NO_3$ pollution of surface and groundwaters. Treatments of fertilizers in combination with nitrification inhibitors have been suggested as a technique to reduce nitrification rate and $NH_3$ volatilization[1,2,3,4]. A nitrification inhibitor may potentially reduce $NO_3^-$ losses due to leaching from $NH_4$-N–liberating fertilizer materials, including organic nitrogen sources, by maintaining nitrogen as $NH_4^+$, which is less susceptible to loss from the soil through this route and through $NH_3$ volatilization[5,6,7,8]. Soil microorganisms are thus of great importance to the nitrogen nutrition of crop vegetation. Such microorganisms are sensitive to changes in the surrounding soil[9,10], and it has been shown that microbial populations change after fertilization[11,12,13]. Fertilizer can directly stimulate the growth of microbial populations as a whole by supplying nutrients, and may affect the composition of individual microbial communities in soil[14,15,16]. The effects of nitrification inhibitor potassium oxalate (PO) on soil microbial populations has, however, not been investigated for Calcisol. The purpose of this study was to investigate the influence of mineral fertilizer combined with PO on the soil microbial population, activities, and nitrification rate in nitrogen-deficient calcareous soil under cotton cultivation in Uzbekistan.

## MATERIALS AND METHODS

### Study Site and Soil Sampling

Sites used in this study represent continuously cultivated (more than 50 years) cotton fields located in Kalinin province northeastern part of Uzbekistan. Soil type is calcareous Calcisol having a calcic horizon within 80 cm of the surface. The orchic horizon is low in organic matter. The climate is semiarid with mean annual air temperatures of 16 and 18°C, and mean annual rainfall of 200 mm. Soil samples were taken from the top 10 cm of soil in an existing cotton field. The cores were pooled; field-moist soils were sieved (<2mm) directly after collection. The soil samples were kept in black polyethylene bags and stored at 4°C. These "fresh" field-moist, sieved samples were used for the incubation study.

### Pot Experiments

Soil microbial activity and nitrogen transformation in soils amended with the mineral fertilizers combined with PO were studied in small pots in laboratory experiments with three replicates. Field-moist subsamples (1 kg) of each treatment replicate were placed in pots and treated with N as urea at a rate of 200 mg $kg^{-1}$, ammophos as P at a rate of 140 mg $kg^{-1}$, and potassium chloride as K at a rate of 60 mg $kg^{-1}$ soil. PO was added to urea and ammophos at a rate of 2%. The control pots ($N_0P_0K_0$) received no PO or fertilizer. Three treatments—$N_{200}P_{140}K_{60}$ (T1), $N_{200PO}P_{140}K_{60}$ (T2), and $N_{200}P_{140PO}K_{60}$ (T3)—were applied for this study. The tested pots were then placed in incubators maintained at 27°C for 45 days.

### Soil Chemical and Physical Analysis

Air-dried samples were analyzed for the total C, N, P, K, and Mg contents. Soil particle distribution was determined using natrium phosphate. The soil chemical and physical properties are presented in Table 1. The total carbon content ($C_{tot}$) was identified by elementary analysis, while total nitrogen content ($N_{tot}$) was determined by the Kjeldahl method. The molybdenum blue method determined the total phosphorus content ($P_{tot}$) in the soil. Potassium (K) was determined using the flame photometric method[17]. The atomic absorption spectrophotometer (AAS) was employed to measure calcium chlorite ($CaCl_2$) and extractable magnesium[18]. Soil pH value was measured by means of an electrometer.

### Soil Microbiological Analyses

After 45 days the pots were removed from the incubation and were analyzed for microbiologic tests. The plate dilution method was used for determination of numerous microorganisms using an agar medium. In order to count the number of microorganisms, 10 g of soil were shaken with 90 ml of ster.-distilled water. From this suspension the serial dilution (1:10) was prepared; plate counts were performed in triplicate and incubated until growth occurred (usually 3 to 7 days). Colony forming units (CFU) of ammonifying bacteria were enumerated on glycerine peptone agar. Mediums containing 10 g of starch, 2 g of $(NH_4)_2SO_4$, 1 g of $K_2HPO_4$, 1 g of $MgSO_4$, 3 g $CaCO_3$, 1 g of NaCI, and 15 g of agar $l^{-1}$ were used for mineral assimilating bacteria. Nitrifying bacteria were determined on plates containing 2 g of $(NH_4)_2SO_4$, 1 g of $K_2HPO_4$, 0.5 g of $MgSO_4$, 0.1 g $FeSO_4$, 5 g $CaCO_3$, and 0.4 g NaCl $l^{-1}$ of liquid medium. Denitrifying bacteria and *Clostridium* were determined on Giltay medium containing 1 g $KNO_3$, 1 g $KH_2PO_4$, 1 g $K_2HPO_4$, 2 g $MgSO_4$, 0.2 g $CaCI_2$, 0.1 mg $FeCI_3$, and 0.1% solution of bromthymol blue. Oligotrophic bacteria were determined on soil agar containing 900 ml water, 100 g soil, and 18 g agar $l^{-1}$. Oligonitrophilic bacteria and *Azotobacter* were determined on Eshbi agar containing 0.2 g $K_2HPO_4$, 0.2 g $MgSO_4$, 0.2 g of NaCI, 0.1 g $K_2SO_4$, 5 g $CaCl_2$, 20 g sacharosa, and agar 15 g $l^{-1}$.

### Soil Biochemical Measurements

Cellulose-degrading activity and amino acid formation of the soil was measured according to the methods in Swyaginzev[19]. Cellulose material was placed into the soil for an incubation period of 45 days. The material was then removed and the cellulose degradation percentage was analyzed. Net nitrifications were measured by incubating the soil samples with the soil moisture content adjusted to 60% of the water holding capacity (WHC), at 28°C for 45 days. The method used is described in detail in Aristowskaya[20]. The data were analyzed using the statistical analysis of variance by outlined in Tepper[21].

**TABLE 1**
**The Soil Chemical and Physical Properties**

| Soil Chemical Properties, mg 100 $g^{-1}$ (0 to 30 cm soil depth) | | | | | | Soil Particle Distribution, % | | |
|---|---|---|---|---|---|---|---|---|
| $C_{tot}$ | $N_{tot.}$ | $P_{tot}$ | K | Mg | pH | Sand | Silt | Clay |
| 200 | 6 | 3 | 12 | 6 | 8.5 | 2.2 | 54.5 | 9.4 |

## RESULTS AND DISCUSSION

### Changes in Soil Microbial Populations

T2 and T3 decreased the number of oligotrophic bacteria compared to the control (Fig. 1). The decrease of colonization frequency of oligotrophic bacteria after inhibitor nitrification in cotton plants has also been reported[6]. Oligotrophic microorganisms are able to survive in nutrient-poor soil; the input of high concentrations of nutrients inhibited their activity**.** The T1 and T2 decreased the number of mineral-assimilating bacteria, while control and ammophos increased the number of these group bacteria (Fig. 1). In particular, decreasing soil water potential following mineral N application and declining pH resulting from nitrification of $NH_4^+$ sources are known to reduce the activity of mineral-assimilating microorganisms[22].

The number of ammonifying bacteria was reduced by T1, T2, and T3 (Fig. 1). This shows the utilization of mineral forms of nitrogen in soil on the background of reducing number of ammonifying bacteria. A decreased number of ammonifying bacteria after application nitrification inhibitor in Calcisol soil has been previously reported[6]. The decrease in microbial indices in the fertilizer treatments could indicate a change in the quality of organic matter to a less available substrate for ammonifying bacteria than in the unfertilized soil[23]. T1, T2, and T3 exhibited an oligonitrophilic bacteria population seven times that of the control (Fig.1). None of the treatments had a negative effect on nitrogen-fixing bacteria *Azotobacter* (data not shown). According to Miyan et al.[24], Kucharski[25], and Govedarica et al.[26] the treatments of nitrification inhibitors also increased the number of oligonitrophilic bacteria and had no negative effects on *Azotobacter.*

The application of fertilizer without nitrification inhibitors had no effect on nitrifying bacteria. Treatments T2 and T3 decreased the number of nitrifying bacteria compared to the control (Fig. 2). The results showed that PO inhibited nitrifiers, which was reflected in the reduction of $NO_3^+$ losses through leaching from fertilizer material. Other authors also reported that Thiourea inhibited the nitrifying activity of nitrifiers, which was reflected in the increased availability and efficiency of fertilizer nitrogen to the rice plants and indicated a potentiality as a nitrification inhibitor[27,28].

The number of denitrifying bacteria in the T2 and T3 samples decreased significantly (by a factor of 25) in comparison with the control (Fig. 2). Denitrifying activity is an indicator of carbon mineralization of soil. Nitrogen fertilization may result in an unbalanced nutrient composition in the soil, which can reduce the denitrifying activity of bacteria. Nitrification inhibitors have a very marked effect on denitrifying microorganisms' production of $N_2$ and $N_2O$ through reduction of $NO_3$ because it blocks reduction of $N_2O$ to $N_2$ by these microorganisms[29]. It has also been found that urea fertilization increases pH value and results in decreased microbial biomass and activity[30].

### Changes in Biochemical Properties

The application of fertilizer increased net nitrification by six times compared to the control (Fig. 3). T2 and T3 decreased the net nitrification compared with fertilizer alone. Nitrification inhibitors reduced the rate of nitrification and so increased the thermal time required for $NH_4$-N depletion and $NO_3$-N accumulation in soil amended with $NH_4$-N–forming materials, compared with fertilizer alone. Some authors suggest the reduction of nitrification after application of nitrification inhibitors[3,8,13]. Our results indicate that PO slows the rate of nitrification and may effectively reduce potential $NO_3^-$ leaching losses.

To assess the potential value of a PO in soil, it is important to have information on other studies concerning the formation of nitrogen in soil. The study of the effect of PO on cellulose degradation activity in soil showed that T1, T2, and T3 increased the cellulose degradation activity of soil, which which indicates an increased number of cellulose-degrading microorganisms (Fig. 4). Other authors also found that after the application of mineral fertilizers, the number of cellulolytic microorganisms became

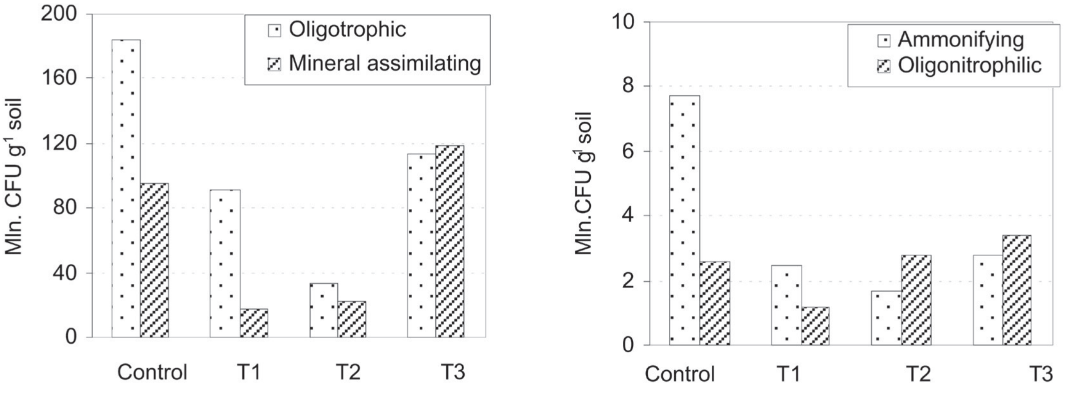

**FIGURE 1.** Influence of mineral fertilizer combined with PO on the number of oligotrophic and mineral-assimilating bacteria (*left*) and ammonifying and oligonotrophilic bacteria (*right*) (T1: $N_{200}P_{140}K_{60}$; T2: $N_{200PO}P_{140}K_{60}$; T3: $N_{200}P_{140PO}K_{60}$).

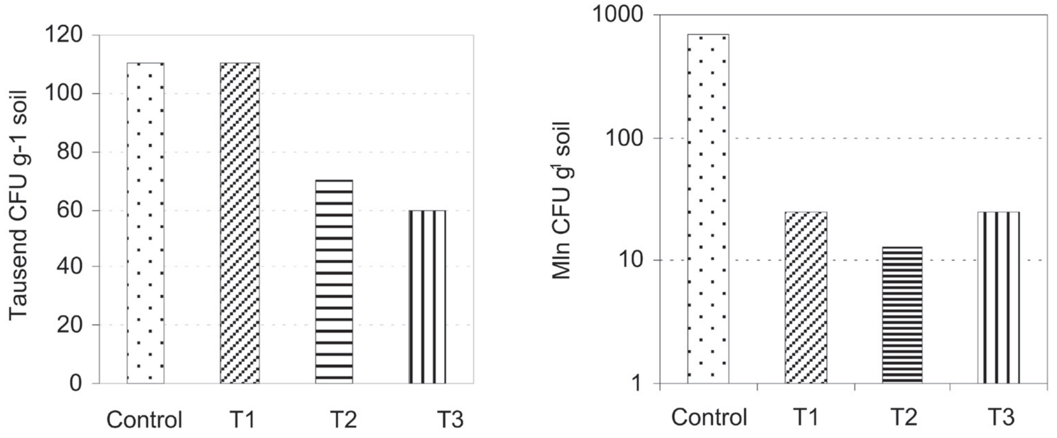

**FIGURE 2.** Effect of mineral fertilizer combined with PO on the number of nitrifying (left) and denitrifying bacteria (right) (T1: $N_{200}P_{140}K_{60}$; T2: $N_{200PO}P_{140}K_{60}$; T3: $N_{200}P_{140PO}K_{60}$).

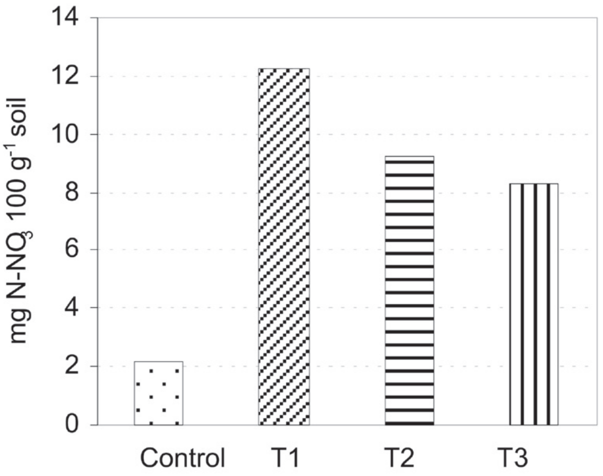

**FIGURE 3.** The effect of mineral fertilizer combined with PO on the net nitrification of soil (T1: $N_{200}P_{140}K_{60}$; T2: $N_{200PO}P_{140}K_{60}$; T3: $N_{200}P_{140PO}K_{60}$).

higher[26]. Additionally, all treatments increased the amino acid formation in the soil (Fig. 4). Similar results were found after the application of nitrification inhibitors AFGUM in Calcisol soil[6].

## CONCLUSIONS

It was clearly demonstrated that fertilization supplemented with a nitrification inhibitor influenced soil microorganisms. All combinations of mineral fertilizers combined with PO during the incubation had an inhibitory effect on the activity of oligotrophic bacteria, ammonifying bacteria, and denitrifying bacteria. The marked stimulus effect on the number of bacteria during the incubation was achieved with T3, the lowest with T2. To summarize, the work reported in this paper suggests that PO combined with mineral fertilizers had no adverse effects on the biological nitrogen fixing bacteria *Azotobacter,* while increasing the activity of oligonitrophilic bacteria, the cellulose degrading activity, and the amino acid formation in soil. PO was indicated potentially as a nitrification inhibitor for the soil of urea used in this study. The treatment T3 decreased the net nitrification compared with fertilizer alone. In conclusion, PO is one of the promising nitrification inhibitor compounds for reducing potential $NO_3^-$ leaching losses from materials caused by nitrifying microorganisms during cotton plant establishment.

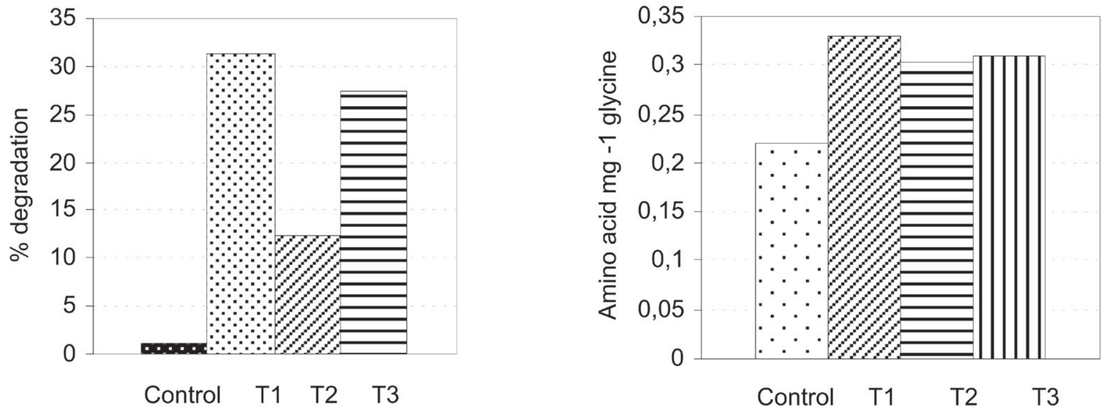

**FIGURE 4.** The effect of mineral fertilizer combined with PO on the cellulose degradation activity (left) and amino acid formation (right) of soil (T1: $N_{200}P_{140}K_{60}$; T2: $N_{200PO}P_{140}K_{60}$; T3: $N_{200}P_{140PO}K_{60}$).

| | | |
|---|---|---|
| Received: | July | 28, 2001 |
| Revised: | October | 10, 2001 |
| Accepted: | October | 15, 2001 |
| Published: | October | 30, 2001 |

## BIOSKETCH

**Dr. Dilfuza Egamberdiyeva** is a Senior Scientist at the Institute of Microbiology, Tashkent, Uzbekistan. She earned her Ph.D. at the Humboldt University, Department of Agriculture and Horticulture, Berlin, Germany in 2000. In 2001 she conducted Postdoctoral research in the Department of Biosciences, Helsinki University, Finland. She has received several awards to attend international meetings. Dr. Egamberdiyeva has published research works in several books, journals, and proceedings, which are printed in European countries. Her research interests include soil microbiology, soil productivity, soil contamination, agricultural management, mineral and organic fertilizers, plant nutrition, and plant productivity.
